# *Streptomyces* spp. as biocatalyst sources in pulp and paper and textile industries: Biodegradation, bioconversion and valorization of waste

**DOI:** 10.1111/1751-7915.14258

**Published:** 2023-04-05

**Authors:** Mara F. Cuebas‐Irizarry, Amy M. Grunden

**Affiliations:** ^1^ Department of Plant and Microbial Biology North Carolina State University Plant Sciences Building Rm 2323, 840 Oval Dr Raleigh North Carolina 27606 USA

## Abstract

Complex polymers represent a challenge for remediating environmental pollution and an opportunity for microbial‐catalysed conversion to generate valorized chemicals. Members of the genus *Streptomyces* are of interest because of their potential use in biotechnological applications. Their versatility makes them excellent sources of biocatalysts for environmentally responsible bioconversion, as they have a broad substrate range and are active over a wide range of pH and temperature. Most *Streptomyces* studies have focused on the isolation of strains, recombinant work and enzyme characterization for evaluating their potential for biotechnological application. This review discusses reports of *Streptomyces‐*based technologies for use in the textile and pulp‐milling industry and describes the challenges and recent advances aimed at achieving better biodegradation methods featuring these microbial catalysts. The principal points to be discussed are (1) *Streptomyces'* enzymes for use in dye decolorization and lignocellulosic biodegradation, (2) biotechnological processes for textile and pulp and paper waste treatment and (3) challenges and advances for textile and pulp and paper effluent treatment.

## Graphical abstract

Members of the genus *Streptomyces* are of interest because of their potential use in biotechnological applications. This review discusses reports of *Streptomyces*‐based technologies for use in the textile and pulp milling industry and describes the challenges and recent advances aimed at achieving better biodegradation methods featuring these microbial catalysts.

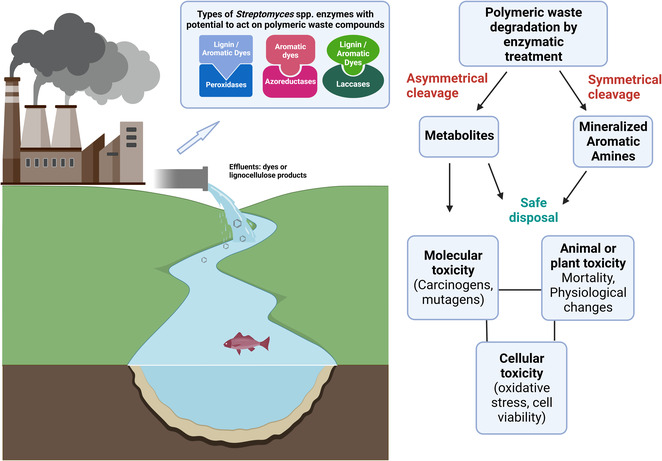

## INTRODUCTION

In recent years, the contribution of wastewater to environmental pollution has been of global concern. The presence of bioactive organic chemicals in water effluents is particularly problematic. Industries such as textile and paper production plants release synthetic dyes and lignin, respectively, as part of their wastewater (Andersson et al., 2021) which could have further negative implications for the environment (Al‐Tohamy et al., 2022; Parmar et al., 2022; Yadav & Chandra, 2018). For example, in paper production, the kraft pulping process is used, which consists of digesting wood under alkaline conditions, followed by several steps of washing and screening to separate brownstock and black liquor (Mathews et al., 2015), a process that generates paper sludge wastewater. The effluents become a collection of fibres, dissolved organic solids, salts, chlorinated compounds, heavy metals and a variety of lignocellulose biomass compounds (Brown et al., 2021), ultimately resulting in both liquid and solid wastes that are of environmental concern (Chandra et al., 2017). They could induce toxicity and endocrine damage if released into the environment (Kumar & Chandra, 2020; Yadav & Chandra, 2018), including negatively impacting aquatic life if introduced into waterways (Singh & Chandra, 2019).

Potential pollutants from these effluents are regulated by environmental agencies in different jurisdictions around the world. For example, the United States Environmental Protection Agency provides a list of pollutants and guidelines for discharge and best management practices to manage effluents and waste streams (US EPA, 2016). In the European Union, there is a ‘Circular Economy Action Plan for a greener and more competitive Europe’ that provides a framework for the prevention of waste, monitoring of toxic substances and the improvement of management practices of secondary raw materials and local waste (European Commission, 2020). However, different countries around the world have varied standards and regulations governing the release of paper and textile industry effluents (Central Pollution Control Board (CPCB), 2000; Meriläinen & Oikari, 2008; Zinabu et al., 2018).

Current treatment approaches for the paper industry effluent have been summarized and are separated into three‐step treatments: physicochemical as the primary treatment (sedimentation, flotation and filtration, oxidation and ozonation); biological as secondary treatment, which is divided into aerobic (activated sludge systems and aerated lagoons) and anaerobic and emerging approaches as tertiary treatments (membrane filtration, adsorption and activated carbon, membrane bioreactors; Kumar, Saxena, et al., 2021). Several investigations, reviews and book chapters have described microbial degradation and decolorization of pulp and paper effluents as promising environmentally friendly technologies that need to be developed and further investigated (Singh et al., 2008; Kumar, Saxena, et al., 2021; Kumar, Srivastava, & Gera, 2021; Kumar et al., 2022).

**FIGURE 1:**
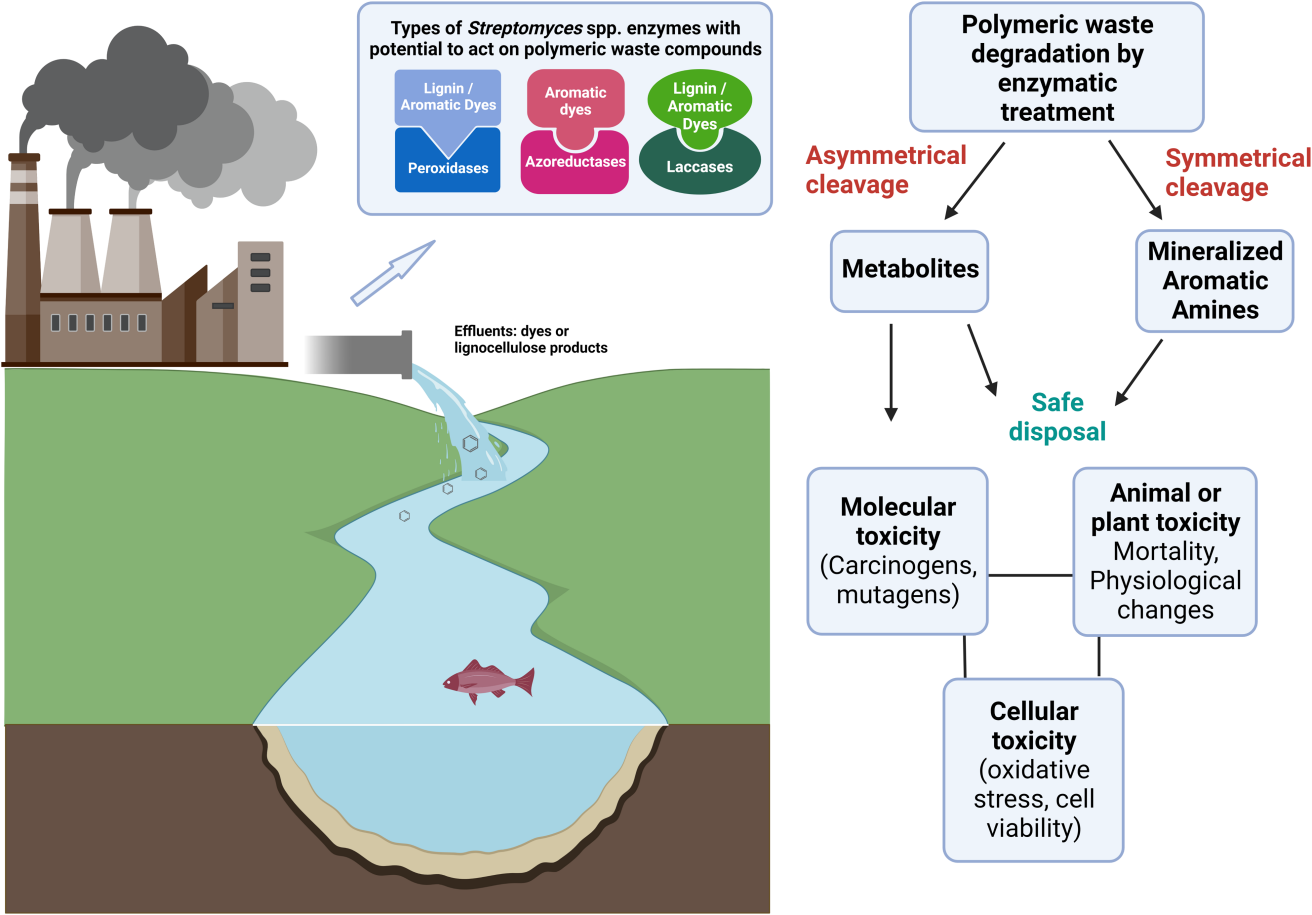
Enzyme types from *Streptomyces* spp. with the ability to degrade pollutants as well as possible degradation by‐products formed and potential effects in the biosphere. Created with BioRender.com.

Effluents from industrial textile operations can contain a range of synthetic dyes mixed with other contaminants at various concentrations (Forgacs et al., 2004; Yaseen & Scholz, 2019). Researchers have reported dye contamination levels of textile and printing effluents ranging from 2% to 50% (w/v; Díez‐Méndez et al., 2019; Tan et al., 2000) and that from 15% to 30% of the dyestuff used in a dye/printing operations may be released to the environment (Bechtold & Turcanu, 2004), while others present information about the types of common dyes that are found in the wastewater effluent (Petrinić et al., 2015). In addition to the textile industry, synthetic dyes are used in pharmaceutical, food, cosmetics, paper, leather and carpet manufacturing (Castro et al., 2021; Guerra et al., 2018; John Sundar et al., 2011; Pérez‐Ibarbia et al., 2016; Saxena et al., 2017). Improper disposal of these dyes represents health risks because of toxic products that biodegradation could produce (Parmar et al., 2022). The increase in fade‐resistant fabric production creates a problem because it uses stable dyes that are more resistant to traditional chemical and biological bioremediation methods (Al‐Tohamy et al., 2022; Carney Almroth et al., 2021). The possible chemical fates of textile and paper pulping effluents based on interactions with enzymes, which can lead to downstream consequences in the environment, are summarized in Figure 1. Currently, decolorization and degradation methods are expensive and sometimes generate toxic compounds that are difficult to degrade (Parmar et al., 2022). Therefore, developing low‐cost, effective bioremediation methods for these colour‐fast dyes is needed.

Lignocellulose is the most globally abundant organic renewable source and can be used as a feedstock to produce sustainable bioproducts (i.e. fuels, chemicals and molecules; Riyadi et al., 2020). Lignocellulose is a complex biopolymer composed of cellulose, hemicellulose and lignin. Lignin, the second most abundant component in lignocellulose after cellulose (Xiao et al., 2020), provides rigidity and robustness to the plant cell wall, notably protecting against pathogens and oxidative stress (Achyuthan et al., 2010; Mathews et al., 2015). However, because of its amorphous structure, lignin can be difficult to break down (Kumar & Chandra, 2020). The removal of lignin is necessary to facilitate bioconversion of the hemicellulose and cellulose components of lignocellulose into sugars for fermentation applications (Jönsson & Martín, 2016; Yu et al., 2018; Zhang et al., 2016) as well as for wood pulp processing for paper production (Wang et al., 2013). Once removed, lignin is often burned to use as steam energy (low‐cost fuel to power paper mills) instead of being reused (Ko et al., 2016). A recent review describes the process of lignin gasification, including the generation of steam energy from lignin residues in the paper pulping process (Castro Garcia et al., 2022). However, the valorization of lignin for its beneficial properties such as biodegradation, antioxidant activity, thermostability, high carbon content and stiffness has been recently reviewed (Rinaldi et al., 2016) as has the bacterial conversion routes for the valorization of lignin (Liu et al., 2022). Challenges remain in the processing of lignin for complete valorization through its bioconversion to bio‐based products (e.g. commodity chemicals like plastics, resins, fibres, bioplastics, resins, fibres and phenols as well as biofuels; Sethupathy et al., 2022). Existing pretreatment methods for lignin degradation may negatively affect downstream stages in energy bioconversion from cellulose biomass and can generate toxic waste products (Klinke et al., 2004; Palmqvist, 2000; Palmqvist & Hahn‐Hägerdal, 2000). Generation of harmful pollutants is a potential risk to the environment and human health (Benslama et al., 2021; Yang et al., 2017). Therefore, treatment options are needed to increase the efficiency of lignin degradation and bioconversion. Using microbial enzymes for lignin and dye degradation is promising for enhancing available treatment options. The purpose of this review is to highlight the ability of multiuse enzymes from a specific bacterial genus, *Streptomyces* spp., to bioremediate textile and pulp‐milling waste streams.

## TAXONOMY AND LIFE CYCLE OF *STREPTOMYCES*

The genus *Streptomyces*, phylum Actinobacteriota (Panda et al., 2022), class Actinobacteria are Gram‐positive, aerobic bacteria with high G + C content (69–78 mol %) that form an extensive mycelium (Barbuto Ferraiuolo et al., 2021; Kämpfer, 2006). *Streptomyces* are ubiquitous in a variety of ecological niches but are commonly found in soil and are ecologically significant due to their role in decomposing cellulose (Takasuka et al., 2013). The genus is arguably most known for secondary metabolite production (Alam et al., 2022; Dávila Costa et al., 2020; Goel et al., 2021, 2022; Kinkel et al., 2014; Lee et al., 2014). *Streptomyces* are well studied as sources for antibiotic production in the pharmaceutical industry (Chevrette et al., 2019). Recent advances in the production of drugs have been reviewed and highlighted as microbial cell factories (Barbuto Ferraiuolo et al., 2021). These microbes contain multiple biosynthetic gene clusters (BGCs) that are sources of bioactive compounds with biomedical and agricultural applications (Nicault et al., 2021; Ward & Allenby, 2018). However, comparably much less discovery has occurred to date focused on developing *Streptomyces‐*based technologies for biodegradation applications.

Physiologically, *Streptomyces* spp. have a life cycle divided into two phases (vegetative and reproductive) that can vary depending on whether the culture is being cultivated on solid or in liquid media (Lajtai‐Szabó et al., 2022). When grown on solid‐based media, the spores germinate into the hyphae that produce the vegetative mycelium. The mycelia grow deeply into the solid media, and some proportion of the mycelia could be present during the whole life cycle (Flärdh & Buttner, 2009). Later, programmed cell death starts a degradation process where the hyphae become multinucleated (Manteca et al., 2005). At this stage, the secondary metabolites are generated, and the aerial mycelia appear. This process is followed by a second programmed cell death event where spore formation occurs (Manteca et al., 2008). If suitable conditions exist to promote the germination of the spores, the cycle starts again (Barbuto Ferraiuolo et al., 2021). When *Streptomyces* are grown in broth cultures, spore germination and mycelium development are similar to growth on solid media; however, differences in morphological characteristics like the propensity for clumping, pellet formation, or having dispersed mycelia can result (Lajtai‐Szabó et al., 2022).

## *STREPTOMYCES* SPP. ENZYMES WITH UTILITY FOR LIGNIN POLYMER MODIFICATION AND DYE DECOLORIZATION AND DEGRADATION APPLICATIONS

Most lignin degradation studies focus on fungal enzymes, suggesting that fungal lignin metabolism is more efficient for lignin degradation than in bacteria (Liu et al., 2022). Fungal species that have been reported to possess lignin depolymerization properties are white‐rot and brown‐rot members, where lignin peroxidase (LiP), manganese peroxidase (MnP), versatile peroxidase (VP), dye‐decolorizing peroxidases (DyP) and laccases (Lac) have been identified and characterized as the key enzymes for this process (Barrasa et al., 1995; de Eugenio et al., 2021; Salame et al., 2014; Salvachúa et al., 2013; Soden et al., 2002). However, the slow growth of fungi makes the process more challenging. Extracellular activity from fungi with the ability to bioremediate dye and lignocellulosic waste has been demonstrated and extensively studied (Ajaz et al., 2020; del Cerro et al., 2021; Fu & Viraraghavan, 2001; Kita et al., 2022). Challenges in improving the expression and activity of fungal enzymes have opened opportunities to investigate the possible use of oxidase‐producing prokaryotic organisms for dye bioremediation (Ajaz et al., 2020). Compared to fungi, bacterial strains are easier to maintain in bioreactor systems. Also, maintaining fungal growth for extended periods (e.g. ≥7 days) is unfavourable for high decolorization rates (Banat et al., 1996; Chang et al., 2004). Bacterial‐based dye bioremediation applications are preferred because prokaryotes are often faster growers and can generate more biomass that can ultimately support higher functional enzyme titers (Kuhad et al., 2012). In addition, bacterial enzymes can exhibit better stability over a range of temperatures, which is important for in situ bioremediation applications (Wang et al., 2022).

Bacterial lignin degradation has not yet been explored to the degree that fungal processes have been studied, but studies have reported lignin‐degrading bacteria in the following classes: Actinobacteria, Alphaproteobacteria, Bacilli and Gammaproteobacteria (Ball et al., 1989; Bugg et al., 2011; Chen et al., 2013; DeAngelis et al., 2011, 2013; Jiang et al., 2022; Manter et al., 2011; Mathews, Grunden, et al., 2016; Mathews, Smithson, et al., 2016; Tian et al., 2014). Genera belonging to these classes are best known for their degradation capabilities because it has been shown through metagenomic analyses and biochemical characterization that several of them possess highly conserved versions of well‐characterized lignin‐degrading enzymes (Tian et al., 2014). The study of enzymatic breakdown of lignin and lignocellulose has fortuitously also resulted in the identification of enzymes capable of decolorizing various chromophores such as those found in industrial dye waste streams (Wang, Li, et al., 2018; Wang, Yao et al., 2018). It is been reported that lignocellulose solubilizing microbes were also capable of the decolorization of triphenylmethane and Poly R‐478 dyes (Abou‐Dobara & Omar, 2015; Ball et al., 1989; Vasdev & Kuhad, 1994). The decolorization process typically occurs through the oxidation of the chromophore portions of dyes or other coloured compounds by enzymes such as laccases, azoreductases and peroxidases (Chen et al., 2003; John et al., 2020; Satheesh Babu et al., 2015).

Several research studies have shown the presence and potential of enzymes from *Streptomyces* spp. for lignocellulose degradation (Blánquez et al., 2017, 2022; Cecchini et al., 2018; Feng et al., 2015; Pinheiro et al., 2017; Riyadi et al., 2020; Takasuka et al., 2013; Ventorino et al., 2016; Wadler et al., 2022), and it has been shown that these microbes produce dye‐decolorizing, detergent‐stable peroxidases that could replace sodium perborate for destaining synthetic textile dyes (Rekik et al., 2015). The presence and/or detected activity of these enzymes in *Streptomyces* spp. supports the idea that this group should be further investigated as a good source of enzymes for lignocellulolytic‐degrading and decolorization applications.

## APPLICATIONS OF *STREPTOMYCES* SPP. IN TEXTILE AND PULP AND PAPER INDUSTRIES

For textile and pulp industries, microbial enzymes have proven valuable to treat water effluents or waste by‐products (Rajoria & Roy, 2022). Because of these potential capabilities, researchers can use different screening methods to discover the presence of relevant enzymes from a variety of environmental samples (Parmar et al., 2022). For example, a dye decolorization screening method was initially used to identify laccases and other enzymes that can break down aromatic compounds (Glenn & Gold, 1983; Ollikka et al., 1993; Pasti & Crawford, 1991). Dye decolorization screens can employ a solid growth media format wherein the breakdown of the dye results in a distinguishable halo surrounding the microbial colony producing the enzyme(s) of interest, or the microbe can be grown in a liquid medium to which a dye has been added. Colour intensity is then quantified using a spectrophotometer (Shah et al., 2021). The dyes used in the screens (Table 1) are complex in chemical composition and utilized for screening methods for these enzymes because they mimic the structural complexity of lignocellulose compounds and can therefore indicate aromatic‐degrading activity (Mathews, Grunden, et al., 2016; Mathews, Smithson, et al., 2016; Tian et al., 2014). These types of plate assays are described in recent reviews as part of the isolation methods for lignin‐modifying enzymes secreted by microorganisms (Kameshwar & Qin, 2017; Kaur et al., 2022).

**TABLE 1:** Dyes of environmental concern in the textile industry and examples of *Streptomyces* spp*.* that have been involved in the treatment of these dyes.

Dye type	Commercial name	Material dye is applied to	Structure	Strains involved in decontamination	References
Acid	Acid blue 74 (Indigo Carmine)	Leather, Wool	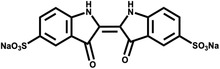	*S. coelicolor*	Dubé, Shareck, Hurtubise, Beauregard, and Daneault (2008), Blánquez et al. (2019)
Azo	Azo Red	Silk, Cotton, Nylon	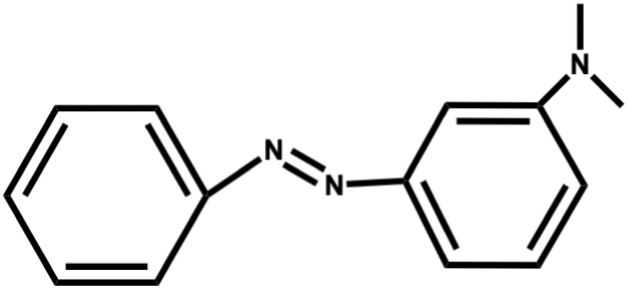	*S*. sp. S27	Dong et al. (2019)
Basic	Methylene Blue	Ink	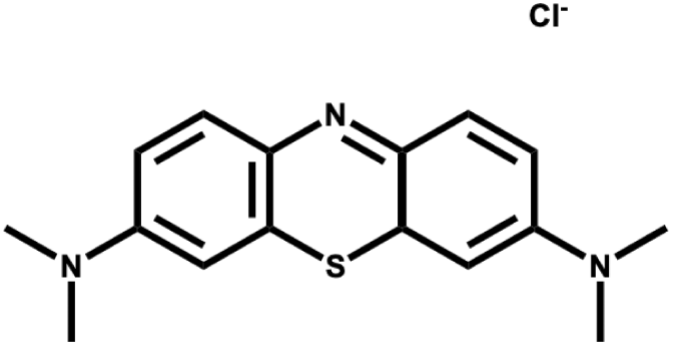	*S. tuirus*	Mechouche et al. (2022)
Direct	Direct sky blue 6b	Leather, cotton, wool, ink	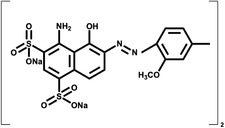	*S. coelicolor*	Dubé, Shareck, Hurtubise, Daneault, and Beauregard (2008)
Sulfur	Thiazine	Cotton, Paper, Silk	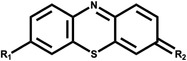	*S. ambofaciens*	Díez‐Méndez et al. (2019)
Reactive	Reactive Black 5	Wool, Silk, Nylon	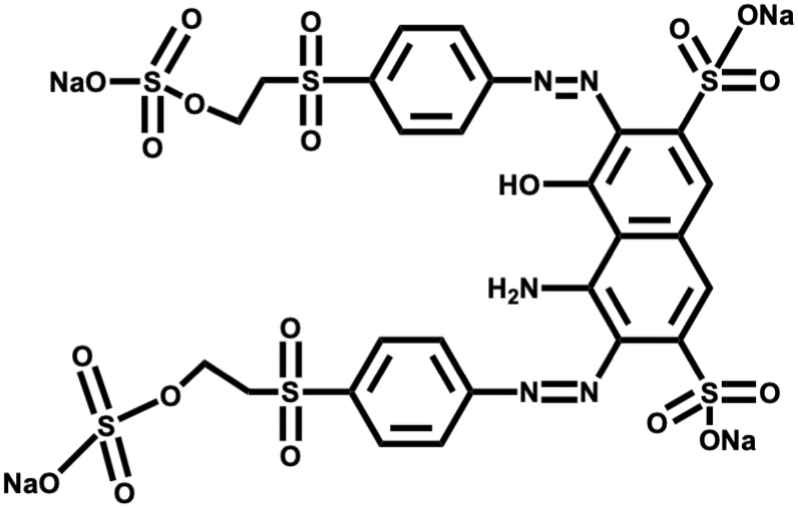	*S. ipomoneae* CECT 3341	Blánquez et al. (2019)
Triphenylmethane	Malachite green, Crystal Violet, Cotton Blue, Methyl Violet	Wool, Silk, Nylon, Cotton	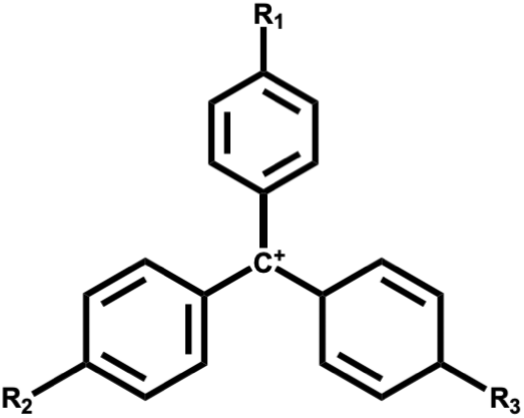	*S*. sp. MN262194	Adenan et al. (2022)

Development of applications using *Streptomyces* to biotransform recalcitrant materials is an area of significant interest. Better understanding of the metabolism of lignocellulosic materials by *Streptomyces* spp. has recently opened a door for the valorization of microbial‐generated products from food waste. For example in a recent, study by Schalchli et al. (2021) *Streptomyces* spp. were used to produce antifungals and biopigments from potato solid waste. Additionally, in the food waste valorization field, an immobilized polygalacturonase from *S. halstedii* ATCC 10897 was used in a bioreactor for the degradation of pear and cucumber residues increasing the sugar content up to 15.33 and 9.35 mg/mL, respectively (Ramírez‐Tapias et al., 2018). Another application for lignocellulosic bioconversion was developed for the generation of branched‐chain fatty acids for lipid‐based biofuel applications where *S. lividans* bioconverted sunflower stalks and rape straw residues into triacylglycerols with a yield of 19%–44% conversion (Dulermo et al., 2016). Therefore, exploring enzymes from *Streptomyces* spp. for potential biotechnological applications such as those discussed here represents an opportunity for both industries to treat polymeric waste material before discharge in both textile and pulp and paper industries. Additional reports and details about the application of *Streptomyces* spp. in these industries are presented in Table 2.

**TABLE 2:** Examples of the applied technologies using *Streptomyces* spp. in pulp, paper and textile industries.

Strain	Application	Industry	Key points	Reference
*Streptomyces*. sp. UAH 15, 23, 30, 51	Decolorization of paper mill effluent	Pulp and Paper	*Streptomyces* isolates were able to decolorize paper mill effluent after pulping wheat straw alkali treatment by 60%–65% Two specific strains (UAH 30, UAH 51) were able to also reduce the molecular weights in high and medium fractions of the effluent	Hernández et al. (1994)
*Streptomyces cyaneus*	Biopulping	Pulp and Paper	The pretreatment of wheat straw in Solid‐State Fermentation improved the strength of the optical and mechanical properties Tear index and burst index were improved (15%, and 19% respectively)	Berrocal et al. (2004)
*Streptomyces drozdowiczii*	Stonewashing and biopolishing	Textile	Cellulase activity worked up to 87% in the presence of commercial detergents and was able to reduce the weight of cotton fabric comparable to commercial treatments (0.75 g vs. 1.12 g from an initial 1.5 g)	Grigorevski de Lima et al. (2005)
*Streptomyces* sp. S27	Bioscouring	Textile	Alkali stable pectate lyase (pH 10) exhibited high activity (>70%) at pH 12.0 and showed similar results in reducing the viscosity of polygaracturonic acid compared to a commercial complex (49% vs. 49.7%) when combined with other enzymes was able to be comparable in bioscouring jute fabric (22.39% vs. 22.99%)	Yuan et al. (2012)
*Streptomyces* sp. NP2, *Streptomyces* sp. NP4	Bio‐colourants	Textile	Isolates were able to generate diffusible deep blue and deep‐red pigments Dyes were tested in polyamide and acrylic fibres with darker colours, polyester and triacetate fibres to a lower dark colour and cotton and cellulosic fibres were weakly coloured Crude bacterial pigments showed similar characteristics of ionic and dispersed dyes due to the polypyrrolic prodigiosin‐like structures that are found in these synthetic dyes	Kramar et al. (2014)
*S*. sp. APL3	Degradation of synthetic polyesters	Textile	*Streptomyces* isolated from compost was able to degrade Polylactic acid, poly‐l‐lactide, polycaprolactone, poly‐(butylene succinate) and polybutylene succinate‐*co‐*adipate	Somyoonsap et al. (2018)
*S. griseorubens* LH‐3	Biobleaching	Pulp and Paper	Purified and characterized endo‐xylanase increased the brightness of eucalyptus kraft pulp by 14.5% and reduced the kappa number by 24.5%	Wu et al. (2018)
*S. sviceus*	Bioremediation of reactive azo dyes	Textile	Optimal decolorization of Congo red‐21 without the release of toxic molecules	Chakravarthi et al. (2020)
*S. rutgersensis* UTMC 2445	Biobleaching	Pulp and Paper	Lignocellulosic soil isolates as sources of enzymes in bleaching applications using xylanases 7% increase in brightness at 30°C, pH 5–7 compared to untreated control 55% of final brightness after biotreatment	Hamedi et al. (2020)
*S. olivaceus* (MSU3)	Biobleaching	Pulp and Paper	Endo‐β‐1,4 xylanase yielded the reduction of sugars of sugars 110 mg/g, the reduction of kappa number of 14.69 *k* and the degree of brightness of 43.62% ISO using sugarcane bagasse pulp Better fibrillation and porosity of pulp than the zinc oxide‐mediated bleaching	Kumar et al. (2020)
*S. cellulosae*	Bioaugmentation	Textile industry	*S. cellulosae* was tested as part of the consortia that accelerated a textile waste compost process by reducing organic matter from 72 to 12 weeks	Biyada et al. (2022)

## OXIDATIVE LIGNIN‐DEGRADING ENZYMES FROM *STREPTOMYCES* SPP. FOR USE IN WASTE DETOXIFICATION

Microorganisms can be used for the detoxification (degradation, decolorization) of industrial waste streams. Figure 1 summarizes several interactions between the chemical structure of the polymer and environmental factors which can lead to downstream consequences in the environment. Microbial enzymes have been shown to degrade synthetic dyes into uncoloured compounds or mineralize them in the environment (dos Santos Bazanella et al., 2013; Khan et al., 2013; Saratale et al., 2011). The loss of colour does not necessarily mean or guarantee that complete mineralization has occurred (Albahnasawi et al., 2020; Franca et al., 2015). One possible fate is the production of amines or intermediates that need to be bioconverted by other microorganisms or methods (Figure 1; Chang et al., 2004). To remove these amines, chemical, physical and biological methods have been used. Examples include activated carbon adsorption, membrane separation, steam distillation, bacterial oxidation, chemical oxidation, electrochemical techniques and irradiation (Bhat & Gogate, 2021; Klibanov & Morris, 1981; Reynolds et al., 2016). Biological methods have been receiving attention because of their low cost as an alternative to chemical and physical removal techniques which are expensive and require time‐consuming analyses (Rathi et al., 2021). However, research in this area is considered to be in the early stages. The primary goal for implementation is to achieve high degradation rates to avoid the transfer of pollutants from the facility to the environment (Rathi et al., 2021).

The ideal scenario is to have microbes with high catalytic versatility to degrade complex mixtures of dyes and that can tolerate harsh conditions such as exposure to detergents, surfactants and metals. Therefore, microbes capable of growing or surviving over a wide range of pH, temperatures and salinity can be helpful for these applications (Anjaneyulu et al., 2005). A summary of enzymes characterized from *Streptomyces* spp. exhibiting lignocellulosic activity and dye decolorization properties are shown in Table 3.

**TABLE 3:** Strains of *Streptomyces* spp. associated with lignocellulose and dye modification.

Strain	Lignin substrate	Dye substrate	Enzyme	NCBI accession numbers of characterized enzymes	Enzyme characterization method	References
*S. flavorivens*	Lignified sclereids				Assayed from supernatant	Sutherland et al. (1979)
*S. badius* ATCC 39117	Labelled lignin	Aromatic dyes	Laccase			McCarthy (1987)
*S. viridosporus* T7A	Kraft lignin, synthetic lignin, 2,4‐diclorophenol (wood preservatives) Wheat Straw	Aromatic dyes	Laccase, H_2_O_2_ peroxidase AliP3		Characterization from crude extract	Yee and Wood (1997), Zerbini et al. (1999), Zeng et al. (2013)
*S.cyaneus* CECT 3335	Kraft pulp biobleaching	Evans Blue dye, Amido Black 10B, Reactive Black 5	Laccase	HQ857207	Ece et al. (2017)	Arias et al. (2003), Moya et al. (2010), Popović et al. (2021)
*S. psammonitcus*		Acid orange, Methyl orange, Bismarck brown	Lignin peroxidase, manganese peroxidase, Laccase		Characterization from crude extract	Niladevi and Prema (2005), Niladevi et al. (2008)
*S. coelicolor*		Direct Sky Blue, Acid blue 74, Reactive black 5	Laccase	CAB45586	Dubé, Shareck, Hurtubise, Beauregard, and Daneault (2008)	Dubé, Shareck, Hurtubise, Beauregard, and Daneault (2008)
*S. coelicolor* A3 (2)	Grass lignocellulose	Brilliant Blue G, and Trypan Blue	Laccase	CAB45586.1	Machczynski et al. (2004)	Majumdar et al. (2014), Yadav and Chandra (2018)
*S. lividans* TK24	Kraft lignin		AliP3	Reported as not available (de Gonzalo et al., 2016)	Wang et al. (1990)	Wang et al. (1990)
*S*. sp. SB086			Laccase		Characterization from crude extract	Fernandes et al. (2014a, 2014b)
*S. griseorubens* JSD‐1	Lignin, Rice straw, Xylan	Indigo carmine				Feng et al. (2015)
*S. griseosporeus* SN9			Lignin peroxidase (LiP‐SN)		Characterization from crude extract	Rekik et al. (2015)
*S. schrestomycetus* S20		Malachite Green				Vignesh et al. (2020)
*S. ipomoea* CECT 3341	Wheat straw	Orange II, Reactive Black 5, Indigo Carmine	Laccase, Laccase‐mediator system	DQ832180.1	Molina‐Guijarro and Pérez (2009)	Molina‐Guijarro and Pérez (2009), Blánquez et al. (2017, 2019)
*S. fulvissimus* CKS7		Crystal Violet				Buntić et al. (2016)
*S. sviceus* SN3		Congo Red	Laccase	SSEG_02446 from CM000951.1	Gunne and Urlacher (2012)	Chakravarthi et al. (2021)
*S. sviceus* KN3		Congo Red, Navy Blue, Textile azo dye effluent		SSEG_02446 from CM000951.1	Gunne and Urlacher (2012)	Chakravarthi et al. (2017)
*S. albus* ATCC 3005	Eucalyptus Kraft pulp, Chlorophenols compounds		Laccase, Lignin peroxidase		Characterization in crude extract	Antonopoulos, Hernandez, et al. (2001), Antonopoulos, Rob, et al. (2001)
*S. coeliflavus* CS‐29		Blue dye 21, and red dye 34	Laccase, Peroxidase, Manganese peroxidase			Mon et al. (2022)
*S. bacillaris*		Malachite Green, Crystal Violet, Cotton Blue, Methyl Violet				Adenan et al. (2022)
*S. sp*. C1		Indigo carmine and diamond black PV	Laccase‐mediator system		Characterization in crude extract	Lu et al. (2013)
*S. thermocarboxydus*	Alkali lignin		MnP‐like, Laccase		Assay detected	Tan et al. (2022)
*S. coelicolor*, *S. griseorubens*, *S.avermitilis*	Wheat bran		Peroxidase in mono and co‐cultures with fungi			Detain et al. (2022)

One of the key enzymes known to be involved in lignin metabolisms is lignin peroxidase (EC 1.11.1.14; LiP) which was first isolated from the fungus *Phanerochaete chrysosporium*, and it was shown that its heme cofactor is required for enzymatic oxidation of aromatic rings (Tien & Kirk, 1984). Because this type of enzyme needs peroxide for activating catalysis, it is called peroxidase. A peroxidase isoform (P3) from *S. viridosporus* T7A was characterized and overproduced in the presence of Acid‐Precipitable Polymeric Lignin (APPL) and was demonstrated to have the ability to oxidize lignin and phenolic compounds, and because of this, it was classified as a lignin peroxidase called ‘Actinomycetes lignin peroxidase’ (ALiP‐P3; Ramachandra et al., 1988; Spiker et al., 1992). This AliP‐P3 could catalyse the oxidation of 2,4‐dichlorophenol in the presence of hydrogen peroxide, which suggested its usefulness for degradation of xenobiotics (Yee & Wood, 1997).

Unfortunately, no protein or gene sequence of the lignin peroxidase was deposited which makes it difficult to compare to other studies for further enzyme characterization (de Gonzalo et al., 2016) of this enzyme (Ramachandra et al., 1988; Thomas & Crawford, 1998; Wang et al., 1990). One of the characterization studies demonstrated that the yeast *Pichia pastoris* can be used as the expression system for recombinant expression of the lignin peroxidase; however, the protein was not purified and biochemically characterized in the study (Thomas & Crawford, 1998). In the same publication, the researchers reported the co‐expression of an endoglucanase gene, suggesting that the lignocellulosic system of *S. viridosporus* was chromosomally clustered (Thomas & Crawford, 1998).

*Streptomyces* spp. such as *S. coelicolor* A3 may also have a role in lignin modification since it has been shown to use grass lignocellulose as a growth substrate and for forming APPL (Majumdar et al., 2014). In light of the importance of LiP enzymes for lignin deconstruction, lignin peroxidase‐like activity has been screened for in other *Streptomyces* sp., for example in *Streptomyces* sp. S6 (Riyadi et al., 2020). However, no homologues or annotated lignin peroxidases have been found in *Streptomyces* that appear to be similar to fungal lignin peroxidases (de Gonzalo et al., 2016; Riyadi et al., 2020). Moreover, homologues of ligninolytic peroxidases have not been extensively studied in bacteria, and no homologues have been found in lignin‐degrading bacteria using gene‐sequencing predictions or proteomes (Davis et al., 2013; de Gonzalo et al., 2016). Reviews about these enzymes called to attention the need for bioprospecting for lignin peroxidases in bacteria because little is known about them (Falade et al., 2017; Lambertz et al., 2016). Recently high LiP activity (1132–2899 U/L) was detected in *Vibrio* strains which could serve as research models for bacterial LiP (Li et al., 2023).

The genome of *Streptomyces* sp. S6 encodes peroxidases with high homology to a different peroxidase family: DyP‐type peroxidases (DyP); however, low activity was observed (Riyadi et al., 2020). Interestingly, in the genome of *S. viridosporous* T7A, an annotated gene encoding for a putative TAT‐secreted DyP (Davis et al., 2013) was hypothesized to be the enzyme that had been described (de Gonzalo et al., 2016). These dye peroxidases (DyP, EC 1.11.1.19) or dye‐decolorizing peroxidases were initially characterized for decolorizing industrial dyes. However, these enzymes act on various substrates, including types of lignin. They differ from typical peroxidases in their substrate preference for anthraquinone dyes and have high peroxidase activity compared to a variety of other organic compounds (Mechouche et al., 2022). Available peroxidases, also utilized for dye decolorization, are heme‐containing proteins that require hydrogen peroxide (H_2_O_2_) or organic hydroperoxides (R‐OOH) to oxidize reducing substrates. The ability to oxidize various substrates makes them applicable for multiple biological processes such as dye decolorization and oxidation of small phenolic compounds including molecules that mimic lignin (Chen & Li, 2016). Peroxidase‐based technology systems are catalytically active at acidic pH from 4 to 6, a limitation for incorporating peroxidases as biocatalysts in detergent formulations (Rekik et al., 2015). The current status regarding the role of bacterial DyP‐type peroxidases in lignin degradation is still under discussion and has recently been reviewed (Sugano & Yoshida, 2021). Interestingly, DyP‐type peroxidase expression has been shown to play a role in life cycle control in *Streptomyces* (Sugano & Yoshida, 2021), and it was demonstrated to participate in switching between vegetative mycelium and aerial hyphae in *S. lividans* (Chaplin et al., 2015).

Another important oxidative enzyme involved in lignin modification and dye decolorization is laccases (EC 1.10.3.2), which were first discovered in the sap of the Japanese lacquer tree (Hoegger et al., 2006; Yoshida, 1883), and are a type of blue polyphenol oxidase belonging to the family of blue multicopper oxidases. These enzymes are encoded in the genomes of fungi, bacteria and animals. A recent review has highlighted information about the mechanism, structure, enzymatic assays, genomic distribution, biotechnological properties and genetic engineering tools including a mention of their use in the pulp, paper, wood and dye/textile industries (Kaur et al., 2022). Laccases from *Trametes versicolor*, a white‐rot fungi, have been demonstrated to exhibit higher activity (20 times greater) compared to *Streptomyces* laccases (Margot et al., 2013). However, *Streptomyces* laccases have shown improved stability over a broader range of temperature, alkaline conditions and salinity, which is helpful for applications in pulp and textile industries for biobleaching and decolorization, respectively (Singh et al., 2011).

Several species of *Streptomyces* are known to express laccases that have been biochemically characterized (Fernandes et al., 2014a, 2014b). Some examples include a laccase from *S. griseus* that has an optimal pH and temperature of 6.5 and 40°C, respectively, and was shown to oxidize *N,N*‐dimethyl‐*p*‐phenylenediamine sulfate (Endo et al., 2003). *S. cyaneus* produces a laccase with an optimal pH and temperature of 4.5 and 70°C, respectively, and reported activity with 2,2′‐azino‐bis(3‐ethylbenzothiazoline‐6‐sulfonate (ABTS)) and 2,6‐dimethoxyphenol (DMP; Arias et al., 2003). The *S. ipomoea* laccase has an optimal pH of 8 and temperature of 60°C when acting on phenolic substrates (Molina‐Guijarro & Pérez, 2009), and *S. sviceus* laccase has an optimal pH of 9 and temperature of 60°C for reactions with DMP and guaiacol (Gunne & Urlacher, 2012). Reports of laccases associated with lignocellulose and dye deconstruction, degradation or decolorization are provided in Table 3.

Polyphenol oxidases (PPO) are members of the multicopper oxidases that share similar catalytic properties to laccases (Janusz et al., 2020). These enzymes require oxygen to catalyse the oxidation of mono‐ and di‐phenols (Sharma & Kuhad, 2009). Interestingly, these enzymes contribute to melanin pigment formation in bacterial cells (McMahon et al., 2007; Wang et al., 2019). The production of melanin by these enzymes has been observed in *Streptomyces* and was correlated with the production of laccase (Claus & Decker, 2006). The activity in bacterial cells and spores suggests a protective role against environmental stress factors such as UV radiation, reactive oxygen species (ROS) and toxic heavy metals (Faccio et al., 2012). In addition, extracellular PPOs could have a role in the polymerization and detoxification of plant phenolic compounds in soil environments (Janusz et al., 2020).

Azoreductases are another type of industrially relevant enzyme that has been reported to be expressed in *Streptomyces* sp. in addition to laccases and peroxidases that have been described earlier. Azoreductases (EC. 1.7.1.6) can be defined as oxidoreductases that are mostly known for the degradation of azo dyes by reducing the azo linkage (‐N=N‐), for example, the reactive dye structures presented in Table 1. These enzymes are categorized into groups according to their cofactor preference (NADH or NADPH) as their electron donor (Dong et al., 2019). A limiting factor in using these enzymes for wastewater treatment is their cofactor requirement as well as their need for redox mediators to facilitate the transfer of electrons from NAD(P)H to the coloured substrate (Mahmood et al., 2016; Verma et al., 2019). However, some research studies have shown that this problem can be overcome by adding coenzymes or integrated enzymatic systems. This was the case for the discovery of a novel azoreductase ‘AzoRed2’ from *Streptomyces* sp. S27 which was used in combination with a glucose‐1‐dehydrogenase from *Bacillus subtilis* to achieve 99% completion of the degradation of the azo dye within 120 min (Dong et al., 2019). Another study involving *S. coelicolor* showed the importance of the azoreductase as the main biocatalyst of decolorization, while the presence of an active DyP‐type peroxidase and laccase played a role in achieving the mineralization of methylene blue in 72 h at 97.5% decolorization (Preethi & Pathy, 2020). Therefore, azoreductases from *Streptomyces* spp. can serve as another potential bioremediation catalyst, but they need to be more systematically explored for their use in wastewater treatment technologies.

## BIOTECHNOLOGICAL APPROACHES USING *STREPTOMYCES* SPP*.* FOR DECONTAMINATION IN TEXTILE AND PULP AND PAPER INDUSTRIES

Bacterial decontamination in general is a promising environment‐friendly and cost‐effective alternative to physiochemical methods that are commonly used (Parmar et al., 2022). The evaluation of these systems requires the analysis of degradation products or metabolites that are produced during the decontamination process. Suitable methods for these analyses can include analytical techniques such as UV–vis spectroscopy, Fourier transform infrared spectroscopy (FTIR), gas chromatography/mass spectrometry (GC/MS), high‐performance liquid chromatography (HPLC), nuclear magnetic resonance spectroscopy (NMR), among others to gain understanding as to the degree of waste compound degradation and compound fate that can be achieved using the microbial catalysts (Saratale et al., 2011). The current approaches for potential decontamination are discussed in the following sections.

### *Streptomyces* whole‐cell technologies

A whole‐cell system screening process is helpful in determining if the microbe can grow and/or metabolize a specific substrate under specific conditions required for degradation processes (Joutey et al., 2013). In *Streptomyces*, whole‐cell applications have been developed that involve native planktonic whole‐cell biocatalysts, immobilized systems and recombinant whole‐cell systems (Salama et al., 2022). Even if the whole‐cell approach might not represent an advantage over using enzymes as catalysts in terms of efficiency, it is often more cost‐effective because protein isolation and purification steps are avoided (de Carvalho, 2016).

Examples of application of this whole‐cell approach for *Streptomyces* bioconversion include the degradation of organophosphorous compounds, which are found in pesticides, using *S. phaeochromogenes* (Santillan et al., 2020). Another study demonstrated that *Streptomyces*. sp. MTCC 7546 could convert acrylonitrile into acrylic acid using both immobilized and planktonic cells (Nigam et al., 2009). The biotransformation of nitriles is important because its toxicity has been associated with cancer and respiratory and neuronal disorders (Ramteke et al., 2013). Recombinant whole cells of *Streptomyces* have been employed to avoid problems that can arise with the use of non‐native systems (e.g. the potential for the formation of aggregated recombinant enzyme inclusions when using *E.coli* expression systems; Salama et al., 2022). For example, *S. lividans* has been extensively studied since it is readily genetically manipulated and can serve as a robust host for recombinant protein expression (Sevillano et al., 2016). In addition, other *Streptomyces* spp. have been studied (Hwang et al., 2021) to determine whether different genetic engineering approaches could be used to efficiently produce secondary metabolites, biosynthetic gene clusters (BGC) and recombinant proteins using genetic modification methods as described in Table 4.

**TABLE 4:** Genomic tools for cloning, assembly and modifying secondary metabolites and recombinant proteins (Updated and modified from Hwang et al., 2021).

Genomic tools	Heterologous host (*Streptomyces* spp.)	References
Genomic Libraries	*S. ambofaciens* BES2074, *S. albus* J1074, *S. coelicolor* M1152, *S. lividans* TK21, *S. coelicolor* strains	Alexander et al. (2010), Li et al. (2017), Tan et al. (2017), D'Agostino and Gulder (2018), Liu et al. (2018), Tu et al. (2018)
Artificial Chromosome Vectors (pSBAC)	*S. lividans* TK21, *S. coelicolor* M145, *S. lividans* K4‐114	Liu et al. (2009), Nah et al. (2015), Luo et al. (2016), Pyeon et al. (2017)
Homologous recombination: Linear‐to‐circular (LCHR), and Linear‐to‐linear (LLHR)	*S. coelicolor* M1146, *S coelicolor* M1152/M1154, *S. venezuelae* WVR2006	Jiang et al. (2013), Yin et al. (2015, 2016), Qian et al. (2020)
Homologous recombination: pSA3239 vector system	*S. lavendulae* subsp. *lavenduale* CCM 3239	Novakova et al. (2022)
In vivo homologous recombination: Exonuclease Combined with RecET recombination (ExoCET)	*S. coelicolor* A3 (2)	Wang, Li, et al. (2018); Wang, Yao et al. (2018)
In vivo homologous recombination: Transformation‐Associated Recombination (TAR)	*S. lividans* TK23, *S. coelicolor* M1146, *S. albus* J1074, *S. albus* TK24, *S. coelicolor* M145	Yamanaka et al. (2014); Bonet et al. (2015); Tang et al. (2015); Bilyk et al. (2016); Novakova et al. (2018)
In vivo assembly: DNA Assembler	*S. lividans* 66, *S. lividans*	Luo et al. (2013), Shao and Zhao (2013), Shao et al. (2013)
Cas9‐ Assisted Targeting of Chromosome segments (CATCH) combined with Gibson Assembly	*S. albus* J1074, *S. avermitilis* MA4680	Jiang et al. (2015), Tang et al. (2018), Tao et al. (2019)

To date, several *Streptomyces* spp. have been identified as sources of enzymes that could be used in dye and lignin degradation either as whole cells or as sources of recombinantly expressed enzymes (Kaur et al., 2022). Vanillin bioconversion to vanillic acid was performed by whole‐cell suspensions of *S. viridosporus* nearly achieving 96% of purity yield (Pometto & Crawford, 1983). A laccase from *S. coelicolor* was recombinantly expressed in *S. lividans* achieving a titre or 350 mg/L with demonstrated activity over a pH range of 4.0–9.0, thermostability up to 70°C, and the ability to decolorize indigo dye in the presence of a mediator system (Dubé, Shareck, Hurtubise, Beauregard, & Daneault, 2008).

### Enzyme mediator systems for *Streptomyces* applications

The lignin degradation process can be improved by the presence of small aromatic molecules called ‘mediators’. In enzymes such as laccases, these mediators can act as electron carriers between the substrate and the enzyme to modulate the redox potential of the reaction system and expand the capacity of the laccase to oxidize structures (Roth & Spiess, 2015). The use of mediators for lignin depolymerization is important since it has been reported that without mediators, laccases can polymerize lignin from small compounds instead of operating in the depolymerization direction (Longe et al., 2018). Furthermore, the polymerization of lignin by a *Streptomyces* laccase has been observed (Majumdar et al., 2014), and it has been observed that a higher reduction of laccases by mediators favours depolymerization, and a lower reduction of laccases in the absence of mediators favours the repolymerization (Chan et al., 2020).

An example of a laccase‐mediator system in *Streptomyces* comes from *Streptomyces* sp. C1. In this case, its small laccase SilA decolorized indigo carmine and diamond black using syringaldehyde as a redox mediator molecule achieving a decolorization of 83.7% and 56.4%, respectively, suggesting that this microbial laccase should be explored for the treatment of textile effluents (Lu et al., 2013). This is a particularly promising application not only because of *Streptomyces* sp. C1 SilA's decolorization properties but also because of its stability under high pH (up to 10) and temperatures (40–50°C; Lu et al., 2013). Several years later, the first example of the use of SilA in a textile dye decolorization application was published, demonstrating that SilA from *S. ipomoneae* CECT 3341 can improve the degradation of textile dyes by up to 60‐fold and 20‐fold, respectively. To achieve these results, a laccase‐mediator system along with acetosyringone and methyl syringate was used (Blánquez et al., 2019). In addition, the same small laccase SilA was recombinantly expressed in *E. coli* and shown to achieve high decolorization levels (more than 80% indigo carmine and malachite green at different pH (6.8–8.0) within 2 h (Coria‐Oriundo et al., 2021)). This study also revealed that β‐(10‐phenothyazyl)‐propionic acid (PhCOOG) could be used as an efficient and affordable mediator in combination with recombinant SilA and could support the decolorization of remanzol brilliant blue R (an anthraquinone dye) by >40% and 80% decolorization of xylidine ponceau (azo dye; Coria‐Oriundo et al., 2021).

For biobleaching applications, *S. cyaneus* 3335 was reported as a source of purified laccase able to catalyse the biobleaching of eucalyptus kraft pulps in combination with 2,2′‐azino‐bis(3‐ethylbenzothiazoline‐6‐sulfonate; ABTS) as a redox mediator (Arias et al., 2003). It was shown to achieve a decrease of 2.3 U in the kappa number, which is a measurement of potassium permanganate solution that is consumed by pulp (Li & Gellerstedt, 1997) and serves as an indication of lignin content or pulp biobleachability. A brightness increase of 2.2% was observed for this study (Arias et al., 2003).

An additional successful laccase‐mediator system was developed using *S. cyaneus* 3335, where the decolorization and detoxification of azo dyes were achieved (Moya et al., 2010). In this case, Methyl Orange and Orange II were decolorized by 90% in the presence of the mediator acetosyringone; however, the viability analyses using *Vibrio fischeri* revealed that toxicity increased by 500% with Methyl Orange and 200% with Orange II after the treatment, while New Coccine and Chromotrope 2R were decolorized and decreased in toxicity by (300 and 185%, respectively, Moya et al., 2010). Based on these findings, it is important to include appropriate toxicological analyses of the treatments and chromatographic analyses for a better understanding of the process. It is also essential to highlight the importance of the cost of laccase‐mediator systems. Mediators can be expensive and incompatible with industrial fermentation processes (Debnath & Saha, 2020). This is a disadvantage that needs to be addressed in future process optimization.

### *Streptomyces* immobilized biocatalyst systems

Enzyme immobilization is also a promising method to consider for textile wastewater treatment. This has been demonstrated in pharmaceutical production (Barbuto Ferraiuolo et al., 2021). Enzyme immobilization can be achieved by any of the following: entrapment, adsorption, binding covalently, self‐immobilization and encapsulation (Fernández‐Fernández et al., 2013). The immobilization technique is favourable to implement since it allows high cell density in the continuous reactors; therefore, the process could be scaled up. Immobilization allows the increment of nutrient availability and consequently, the improvement of catalytic activity and substrate uptake (Rajoria & Roy, 2022).

Recently, it was shown that an immobilized laccase from *S. sviceus* KN3 could successfully mineralize and detoxify Congo Red (azo dye; Chakravarthi et al., 2021). In this study, the crude laccase extract was able to decolorize Congo Red by 69% after 48 h, while the purified and immobilized version showed a decolorization of 78% and 92% after 24 h, respectively. Another immobilized laccase method using *S. cyaneus* 3335 was evaluated for the decolorization of Amido Black 10B (61% decolorization), Reactive Black 5 (100% decolorization), Evans Blue (90% decolorization) and Remanzol Brilliant blue (100% decolorization; Popović et al., 2021). This study demonstrated that immobilized laccases from *S. cyaneus* 3335 can effectively catalyse delignification in a biobleaching process of paper pulping (Popović et al., 2021).

### Application of *Streptomyces* consortia

Most of the dye degradation studies featuring *Streptomyces* as a biocatalyst source describe the utility of recombinant enzymes or whole‐cell catalysts in pure culture. To maximize decolorization or degradation, consortia studies have also been performed. A *Streptomyces* consortium was used to biodegrade Reactive Blue 222, a reactive sulfonated di azo dye (Pillai, 2018). This process enhanced the biodegradation of the dye by 89% using two different *Streptomyces* spp. This rationale for microbial consortium degradation of waste products has been seen using *Streptomyces* spp. along with yeast species to bioconvert lignocellulosic components into biofuels (Wadler et al., 2022), where it was observed that some individual strains were only able to use about 40% of the soluble lignocellulosic components, while the mixed consortia increased the degradation of the soluble lignocellulosic components by up to 70%.

### Bioreactors

Optimization studies using bioreactors have been employed to evaluate the proposed biodegradation of waste at an industrial scale (Barbuto Ferraiuolo et al., 2021; Buntić et al., 2016; Olmos et al., 2013). The optimization of laccase production from Actinobacteria was performed with *S. psammoticus* with different scale‐up strategies, resulting in 215.6 U/g as the best yield in the presence of mediators to achieve decolorization of azo dyes (Niladevi et al., 2008; Niladevi & Prema, 2005). The peroxidase‐like activity production of *Streptomyces*. sp. strain BSII#1 was scaled up to 3 L culture volumes with an airlift bioreactor achieving 4.76 U/mL in the presence of veratryl alcohol as an inducer. Bioreactor‐based degradation of xylene, a toxic aromatic compound, by *S*. sp. AB1 was investigated, and it was shown that the biocatalyst was able to achieve high elimination levels (90%) of xylene from contaminated water (Chikhi et al., 2016).

Solid‐state fermentation has been reported as an ecofriendly tool in the biopulping process as a method for biological treatment of the wheat straw using *S. cyaneus* (Berrocal et al., 2004). It was also shown that this microorganism was able to increase acid‐soluble lignin from wood chips by reducing the energy required for the process by 24% (Hernández et al., 2005). Furthermore, *Streptomyces* sp. MDG147 has been employed for the valorization of soda lignin from wheat straw to produce oleogels which are used in lubricant applications (Borrero‐López et al., 2018). The same strain, along with MDG301 was also used for the valorization of agricultural residues (barley and wheat straw) to generate APPL and alkali lignin, which after soda pulping exhibited adhesive properties (Blánquez et al., 2022).

## CONCLUSIONS AND FUTURE DIRECTIONS

The textile and paper production can generate hazardous waste containing dyes from the industrial processing of synthetic and natural polymers. Therefore, safe degradation or decolorization applicable to industrial residues is needed (Saxena & Bharagava, 2020). *Streptomyces* spp. offer a valuable source for generating effective enzyme formulations and the development of large‐scale methods for microbial‐based waste treatment. Identifying methods as novel alternatives requires expanding the available enzymatic repertoire and understanding the mechanisms for degradation and bioconversion. Improvements in existing and forthcoming technologies can be realized through rigorous biochemical characterization of enzymes of interest, expansion of the enzymatic repertoire for biodegradation, and through evaluation of application scalability and reproducibility.

Immobilized *Streptomyces* enzymes are promising for large‐scale waste treatment. However, research is needed to apply those technologies to other biotechnological processes. To do so, it is necessary to understand the microbial mechanisms employed by the potential biocatalysts for waste compound degradation. Metabolomic and biochemical insights generated from studies of degradation, decolorization and bioconversion need to be coupled to better understanding of the microbial metabolism that controls the biochemical processes. A way to advance a systematic understanding of the underlying microbial metabolic processes is through the implementation of functional ‐omics (e.g. transcriptomics, metatranscriptomics, genomics, proteomics and metagenomics) in conjunction with the waste compound degradation studies. These analysis approaches could be applied to the examination of pure cultures as well as when evaluating consortia. In addition, the design of mutagenesis experiments including genome editing and directed evolution could add to the understanding of the physiology and mechanisms of biodegradation, which is key to improving the application development for scalable use in both industries.

A strong case has been made that *Streptomyces* are suitable sources of enzymes that could be used for waste treatment. The main challenge remains in the development of large‐scale production methods which can be achieved through a combination of strategic genetic engineering of *Streptomyces* biocatalysts to improve enzyme expression, activity, and robustness and by using our growing understanding of *Streptomyces* physiology to enhance growth and biocatalyst performance.

## AUTHOR CONTRIBUTIONS

**Mara F. Cuebas‐Irizarry:** Conceptualization (equal); writing – original draft (equal). **Amy M. Grunden:** Conceptualization (equal); project administration (equal); resources (lead); supervision (lead); writing – review and editing (lead).

## CONFLICT OF INTEREST STATEMENT

Mara F. Cuebas‐Irizarry declares that she has no conflict of interest. Amy M. Grunden declares that she has no conflict of interest.

## ETHICS STATEMENT

This review article does not contain studies with human participants or animals conducted by any of the authors.
